# Determination of the Cell Permissiveness Spectrum, Mode of RNA Replication, and RNA-Protein Interaction of Zika Virus

**DOI:** 10.1186/s12879-017-2338-4

**Published:** 2017-03-31

**Authors:** Wangheng Hou, Najealicka Armstrong, Lilian Akello Obwolo, Michael Thomas, Xiaowu Pang, Kevin S. Jones, Qiyi Tang

**Affiliations:** 1 Department of Microbiology, Howard University College of Medicine, Seeley Mudd Building, Room 315, 520 W Street, NW, Washington, DC 20059 USA; 2 Department of Oral Pathology, Howard University/College of Dentistry, Washington, DC 20059 USA; 3 Department of Biology, Howard University, Washington, DC 20059 USA

**Keywords:** Zika virus (ZIKV), RNA in situ hybridization (RISH), Permissiveness, RNA Chromatin Immunoprecipitation (ChIP), RNA replication

## Abstract

**Background:**

Two lineages of Zika virus (ZIKV) have been classified according to the phylogenetic analysis: African and Asian lineages. It is unclear whether differences exist between the two strains in host cell permissiveness, this information is important for understanding viral pathogenesis and designing anti-viral strategies.

**Methods:**

In the present study, we comparatively studied the permissive spectrum of human cells for both the African (MR766) and Asian strains (PRVABC59) using an RNA in situ hybridization (RISH) to visualize RNA replication, an immunofluorescence technology, and a western blot assay to determine viral protein production, and a real-time RT-PCR to examine viral RNA multiplication level. The experiments were undertaken in the condition of cell culture.

**Results:**

We identified several human cell lines, including fibroblast, epithelial cells, brain cells, stem cells, and blood cells that are susceptible for the infection of both Asian and African strains. We did not find any differences between the MR766 and the PRVABC59 in the permissiveness, infection rate, and replication modes. Inconsistent to a previous report (Hamel et al. JVI 89:8880–8896, 2015), using RISH or real-time RT-PCR, we found that human foreskin fibroblast cells were not permissive for ZIKV infection. Instead, human lung fibroblast cells (MRC-5) were fully permissive for ZIKV infection. Surprisingly, a direct interaction of ZIKV RNA with envelop (E) protein (a structure protein) was demonstrated by an RNA chromatin immunoprecipitation (ChIP) assay. Three binding sites were identified in the ZIKV RNA genome for the interaction with the E protein.

**Conclusion:**

Our results imply that the E protein may be important for viral RNA replication, and provide not only the information of ZIKV permissiveness that guides the usage of human cells for the ZIKV studies, but also the insight into the viral RNA-E protein interaction that may be targeted for intervention by designing small molecule drugs.

**Electronic supplementary material:**

The online version of this article (doi:10.1186/s12879-017-2338-4) contains supplementary material, which is available to authorized users.

## Background

Zika virus (ZIKV) has recently drawn worldwide attention due to the recent outbreaks that are temporally and spatially consistent with the increased occurrence of congenital microcephaly and Guillain–Barré syndrome (GBS) in the Americas. Recently, an increasing number of strains of ZIKV have been isolated from more than 60 countries since its first isolation in Uganda in 1947 [1, 2]. Phylogenetic studies classified ZIKV into Asian and African lineages [3, 4]. For unknown reasons, the Asian lineage ZIKV has been linked to recent epidemics and most likely the congenital microcephaly and GBS while African strains cause milder symptoms. The recent cases of microcephaly and GBS linked to ZIKV are mostly, if not all, caused by the strains of Asian lineage [5]. However, more experimental evidence is needed to support the hypothesis that ZIKVs from the two lineages are different in viral replication, pathogenesis, transmission, and cell permissiveness.

The first ZIKV was isolated from a monkey, and it is known that ZIKV can be transmitted by being bitten by the infected *Aedes* species of mosquito [6, 7] or sexually between humans [8, 9]. The known primary hosts of ZIKV include human, monkey, and mosquito. During the evolution of ZIKV, the virus may have developed new molecular relationships with factors of the host cells. Only a few human cells are known to be permissive for ZIKV replication including an epithelial cell line (A549), neural stem cells [10], and a skin fibroblast cell line [11]. It remains unknown whether other cell lines are permissive for the infection of ZIKV.

Little is known regarding the interaction of ZIKV proteins and RNA with the host or viral factors although the interactions may determine the fate and/or efficiency of infection, pathogenicity, transmission, and epidemic potential of the ZIKV. It therefore remains important to determine the spatial relationship between the viral proteins and RNA replication. Of equal importance is their temporal relationship, whether the viral RNA replication occurs before protein production.

Belonging to family *Flaviviridae*, ZIKV contains a positive single stranded RNA (ssRNA) genome with a size around 11 k nucleotides (nt). After infecting permissive cells, the ZIKV genome is translated into a precursor protein (a polyprotein) at a size of about 3424 amino acid (Aa). The precursor protein is then co- and post-translationally processed by viral and cellular proteases into 3 structural and 7 non-structural (NS) proteins. Viral replication has been demonstrated in mitochondria and endoplasmic reticulum (ER) [12]. Viral RNA replication has not been characterized for ZIKV. In the present study, we determined the viral permissiveness in different human cell lines using anti-viral antibody, real-time RT-PCR, and RNA in situ hybridization (RISH), then we examined the interaction between the viral RNA and E protein by an RNA ChIP (Chromatin Immunoprecipitation) assay.

## Methods

### Cell lines, Tissue Culture, and Viruses

To examine the spectrum of the permissiveness of ZIKV in cells, we infected different cell lines listed in Table 1 where the sources and types of the cells are indicated. The cells were maintained in the Minimum Essential Medium Eagle (MEM, Sigma M4655) supplemented with 10% fetal calf serum (FCS) and penicillin (100 IU/ml)-streptomycin (100 μg/ml) and amphotericin B (2.5 μg/ml). The peripheral blood mononuclear cell (PBMC), CEM T cells, and THP-1 monocytes were cultured in RPMI 1640 supplemented with 10% fetal calf serum (FCS) and penicillin (100 IU/ml)-streptomycin (100 μg/ml) [13]. ZIKV strains MR766 [14] and PRVABC59 [4] were obtained from ATCC.

**Table 1:** Cell Permissiveness of Zika Virus (ZIKV)

Host cell infected	ZIKV (MR766)		ZIKV (PRVABC59)	
	Protein	RNA	Protein	RNA
Human fibroblast cells				
HFF (ATCC® SCRC-1041™)	−	−	−	−
MRC-5 (ATCC® CCL-171™)	++++	++++	++++	++++
BJ (ATCC® CRL-2522™)	+/−	−	+/−	−
Human Epithelial cells				
ARPE-19 (ATCC® CRL-2302™)	+	+	+	+
A549 (ATCC® CCL-185™)	++	++	++	++
HT1080 (ATCC® CCL-121™)	+/−	+/−	+/−	+/−
Hep-2 (ATCC® CCL-23™)	−	−	−	−
HEK 293T(ATCC® CRL-1573™)	+	+	+	+/−
Human Endothelial cells				
SLK (AIDSRP, cat# 9402)	−	−	−	−
Human blood cells				
CEM/CD4 T cell (AIDSRP, cat# 117)	+/−	+/−	+/−	+/−
THP-1 (AIDSRP, cat# 9949)	−	−	−	−
PBMC (ATCC® PCS-800-011™)	++	++	++	++
Other Human cells				
U-251MG (SIGMA 09063001)	++++	++++	++++	++++
Neural Stem cell	+++	+++	+++	+++
SK-N-SH (ATCC® HTB-11™)	++++	++++	++++	++++
Simian cells				
Vero (ATCC® CCL-81™)	++++	++++	++++	++++
COS7 (ATCC® CRL-1651™)	++++	++++	++++	++++
Mouse cells				
MEF	−	−	−	−
NIH3T3 (ATCC® CRL-1658™)	−	−	−	−

The cells were infected with ZIKV at an MOI of 1 for 48 h, and fixed to perform immunostaining using anti-ZIKV antibody and RISH (RNA in situ hybridization) using the probe against ZIKV genome. Total cells (DAPI) and positive cells (ZIKV protein and/or RNA replication) were counted to calculate the positive rate (Positive rate = positive cell counted / total cells counted). *HFF* human foreskin fibroblast, *MEF* mouse embryo fibroblast. -: less than 3%; +/−: 3–5%; +: 5–10%; ++: 10–30%; +++: 30–70%; More than 70% positive rate defines ++++. *AIDSRP* AIDS Reagent Program

### Antibodies

Anti-Giantin (ab24586) for visualizing Golgi body, anti-Cox IV (ab16056) for showing mitochondria, and anti-Calreticulin (ab196156) for examining endoplasmic reticulum (ER) were purchased from Abcam (Cambridge, MA). The anti-ZIKV envelope antibody was generated from the hybridoma cell line, D1-4G2–4-15 (ATCC® HB-112™) and anti-ZIKV serum was produced from ZIKV-infected mice in our laboratory.

### Western blot assay

Viral and cellular proteins in the whole cell lysate (WCL) samples were separated by 7.5% sodium dodecyl sulfate-polyacrylamide gel electrophoresis (10 to 20 μg loaded in each lane using Novex NuPAGE SDS-PAGE Gel System purchased from ThermoFisher Scientific.), transferred to nitrocellulose membranes (Amersham Inc., Piscataway, NJ), and blocked with 5% nonfat milk for 60 min at room temperature. Membranes were incubated overnight at 4 °C with primary antibody followed by incubation with a horseradish peroxidase-coupled secondary antibody (Amersham Inc.) and detection with enhanced chemiluminescence (Pierce, Rockford, Ill.), according to standard methods. Membranes were stripped with stripping buffer (100 mM β-mercaptoethanol, 2% SDS, 62.5 mM Tris-HCl, pH 6.8), washed with 0.1% PBS-Tween 20, and used to detect additional proteins.

### RNA isolation and real-time RT-PCR

Following instructions of the manufacturers, total RNA was isolated using Aurum™ Total RNA Mini Kit (Bio-Rad, Cat# 732–6820). To quantitatively examine the RNA level of ZIKV from the infected cells, real-time RT-PCR was undertaken using the SsoAdvanced™ Universal SYBR Green Supermix kit (Bio-Rad, Hercules, CA). The primers for ZIKV were: forward- 5′-AAATACACATACCAAAACAAAGTGGT-3′ and reverse- 5′-TCCACTCCCTCTCTGGTCTTG-3′; and the primers for beta-actin (as control) were: forward- 5′ -GGTTCCGATGCCCTGAGGCTC-3′ and reverse- 5′-ACTTGCGGTGCACGATGGAGG -3′. 0.5–2 μg of total RNA and 250 nM of sense and antisense primers (amplifying the RNA fragment in NS5 location) were used in a final 10 μl volume containing 100 ng of cDNA for all samples except for PBMC, which had 25 ng of cDNA. PCR reactions consisted of 40 cycles with the following optimal conditions: 95 °C for 30 s followed by a two-step PCR reaction of 95 °C for 15 s and 60 °C for 30 s. All samples were run in technical triplicates, and the data were collected and recorded by the CFX Manager software (Bio-Rad). The data was analyzed using 2^ΔΔCq^ = (C_q,Target_-C_q,Actin_)_Time X_ - (C_q,Target_-C_q,Actin_)_Time 0_ to obtain the fold change in expression via the 2^ΔΔCq^ method Time 0 was used as the calibrator of the relative quantification of product generated in the exponential phase of the amplification curve for real-time RT-PCR. A melting temperature curve analysis was obtained by measuring (after the amplification cycles) the fluorescence during a period of warming from 65 to 95 °C.

### RNA Chromatin immunoprecipitation (ChIP) assay

The ChIP assay was carried out according to the manufacturer’s manual using an EZ ChIP kit purchased from Millipore (Temecula, CA). Briefly, Vero cells were infected with ZIKV MR766 for 24 h. The cells were cross-linked with 1% paraformaldehyde and then sonicated to shear the DNA/RNA. The RNA-protein complexes were pulled down by anti-ZIKV serum that was generated and purified in our laboratory and normal mouse IgG (used as negative control). The cross-linked RNA-protein complexes were then washed with multiple buffers (provided by the EZ ChIP kit) and reversed by SDS at 95 °C; the DNA was removed by DNase I digestion, the proteins were removed by proteinase K, and the RNA was purified through the provided column. Real-time RT-PCR was performed to examine the amount of RNA in each sample using the primers shown in Table 2.

**Table 2:** Primers for the ZIKV RNA ChIP assays

Sequence	Start nt	End nt
Forward AGTTGTTGATCTGTGTGAGTCAG	1	23
Reverse GCATATTGACAATCCGGAATCCT	133	156
Forward GATTCCGGATTGTCAATATGCTAAA	135	160
Reverse GTGATGGCTTGATTGCTGTAAA	272	294
Forward AGCCATCACTGGGCCTT	285	302
Reverse TAGTCAGCAGGAGGCCAAT	443	462
Forward TCCTGCTGACTACAGCCAT	450	469
Reverse TGCCCGAGGTCCATGAT	584	601
Forward CAAGTGCCACGTACAGATCAT	568	589
Reverse TAGATCGCCGTGCCTCA	730	747
Forward GCACGGCGATCTAGAAGAG	734	753
Reverse AATGGCAACGGCCACTA	882	899
Forward TAGTGGCCGTTGCCATTG	882	900
Reverse AAGACAACATCAACCCAGGTC	1030	1051
Forward TGGGTACCAACTGGGAGAA	10,031	10,050
Reverse TCCTAGATAGGGAATGTCTGTCC	10,170	10,193
Forward AAATGGACAGACATTCCCTATCT	10,166	10,189
Reverse TAGCGGACTTGGGTGGATA	10,323	10,342
Forward TATCCACCCAAGTCCGCTAC	10,323	10,343
Reverse CGTTCTCGGCCTGACTATGA	10,475	10,495
Forward CTCATAGTCAGGCCGAGAAC	10,474	10,494
Reverse CACAGCTAGTCTCCAGTTCAG	10,623	10,644
Forward GAACTGGAGACTAGCTGTGAAT	10,625	10,646
Reverse GCTGTTCGGCGATCTGT	10,760	10,776

Based on the nt sequence of MR766

### Immunocytochemistry and fluorescence in situ hybridization

Cells grown on coverslips, and immunostaining was performed after fixation with 1% paraformaldehyde (10 min at room temperature) and permeabilization in 0.2% Triton (20 min on ice) by sequential incubation with primary and Texas red (TR) -labeled secondary antibodies (Vector Laboratories, Burlingame, Calif.) for 30 min each (all solutions in PBS). For simultaneous detection of ZIKV RNA, cells were first immunostained for cellular or viral proteins and then treated for 1 h at 37 °C with RNase-free DNase I (Roche, Indianapolis, Ind.; 200 U/ml in PBS containing 25 mM MgCl2). After refixation in 4% paraformaldehyde (10 min at room temperature), samples were equilibrated in 2× SSC (1× SSC is 0.15 M NaCl plus 0.015 M sodium citrate), dehydrated in ethanol (70, 80, and 100% ethanol for 3 min each at −20 °C), air dried, and incubated overnight at 37 °C with the hybridization mixture. To detect RNA, only the probe DNA was denatured at 94 °C for 5 min. After hybridization, samples were washed at 37 °C with 55% formamide in 2× SSC (twice for 15 min each), 2× SSC (10 min), and 0.25× SSC (twice for 5 min each). Hybridized probes were labeled with FITC-avidin (Vector Laboratories; 1:500 in 4× SSC plus 0.5% BSA), and signals were amplified by using biotinylated anti-avidin (Vector Laboratories; 1:250), followed by another round of FITC-avidin staining. Finally, cells were equilibrated in PBS, stained for DNA with Hoechst 33,258 (0.5 μg/ml), and mounted in Fluoromount G (Fisher Scientific, Newark, Del.).

### Probe preparation-Nick translation

The plasmid pZIKVMR766 containing the whole ZIKV cDNA was used to be labeled with biotin-11-dUTP by nick translation. The DNase concentration for nick translation was adjusted to yield probe DNA 200 to 500 bp in length. Probe DNA was dissolved at 10 ng/μl in Hybrisol VII (Oncor, Gaithersburg, Md.) containing 100 ng of salmon sperm DNA (Gibco-BRL), 1 μg of yeast tRNA (Sigma), and 0.5 mg of cot1 DNA (Gibco-BRL)/μl.

### Confocal microscopy

Cells were examined with a Leica TCS SPII confocal laser scanning system. Two or three channels were recorded simultaneously and/or sequentially and controlled for possible breakthrough between the fluorescein isothiocyanate and Texas Red signals and between the blue and red channels.

## Results

### ZIKV MR766 and PRVABC59 have a similar permissiveness spectrum in human cell lines

Phylogenetic studies based on viral nucleotide and amino acid sequences classify Zika viruses into African and Asian lineages [3, 4, 15]. ZIKV MR766 was isolated from Uganda in 1947 and is the representative of African strains [14]. PRVABC59 strain was isolated in 2015 from a Puerto Rican patient who was infected by a Brazilian strain [4]. According to the phylogenetic studies, PRVABC59 stands for an Asian strain [4]. We were curious about whether they have a different permissiveness spectrum in cell lines of human, monkey and mouse.

Several methods were employed to determine the permissiveness of ZIKV in different types of cell lines as listed in Table 1. First, we performed immunofluorescence assay (IFA) combined with RNA in situ hybridization (RISH). We infected cells with MR766 or PRVABC59 (PR) for 24 h at an MOI of 0.5. After fixation with 1% paraformaldehyde, the cells were permeabilized and immunostained for viral E protein with anti-E protein antibody in red. After refixation with 4% paraformaldehyde, the cells were hybridized with biotin-labeled DNA probe made by Nick translation [16] from a plasmid carrying the whole DNA derived from ZIKV genomic cDNA that was synthesized by the Genescript Inc. (Piscataway, NJ). The RNA was stained in green with FITC-avidin. The cell nuclei were shown in blue using DAPI staining. If the infection rate was greater than 70%, it is set as “++++” in both IFA (red) and RISH (green). The method for defining the infection rate was referenced to that used for cytomegalovirus infection [17] and described in the legend of Table 1.

First, as shown in Additional file 1: Figure S1, we infected Vero cells with MR766 strain or PRVABC59 strain at the same MOI (0.5) for 24 h. The cells were fixed for immunostaining with anti-E protein in red and RISH for RNA in green. After repeating the experiments independently for 3 times, as can be seen in the Additional file 1: Figure S1, there is no differences regarding the infection rate, RNA replication pattern and E protein production between the two strains.

**Fig. 1:**
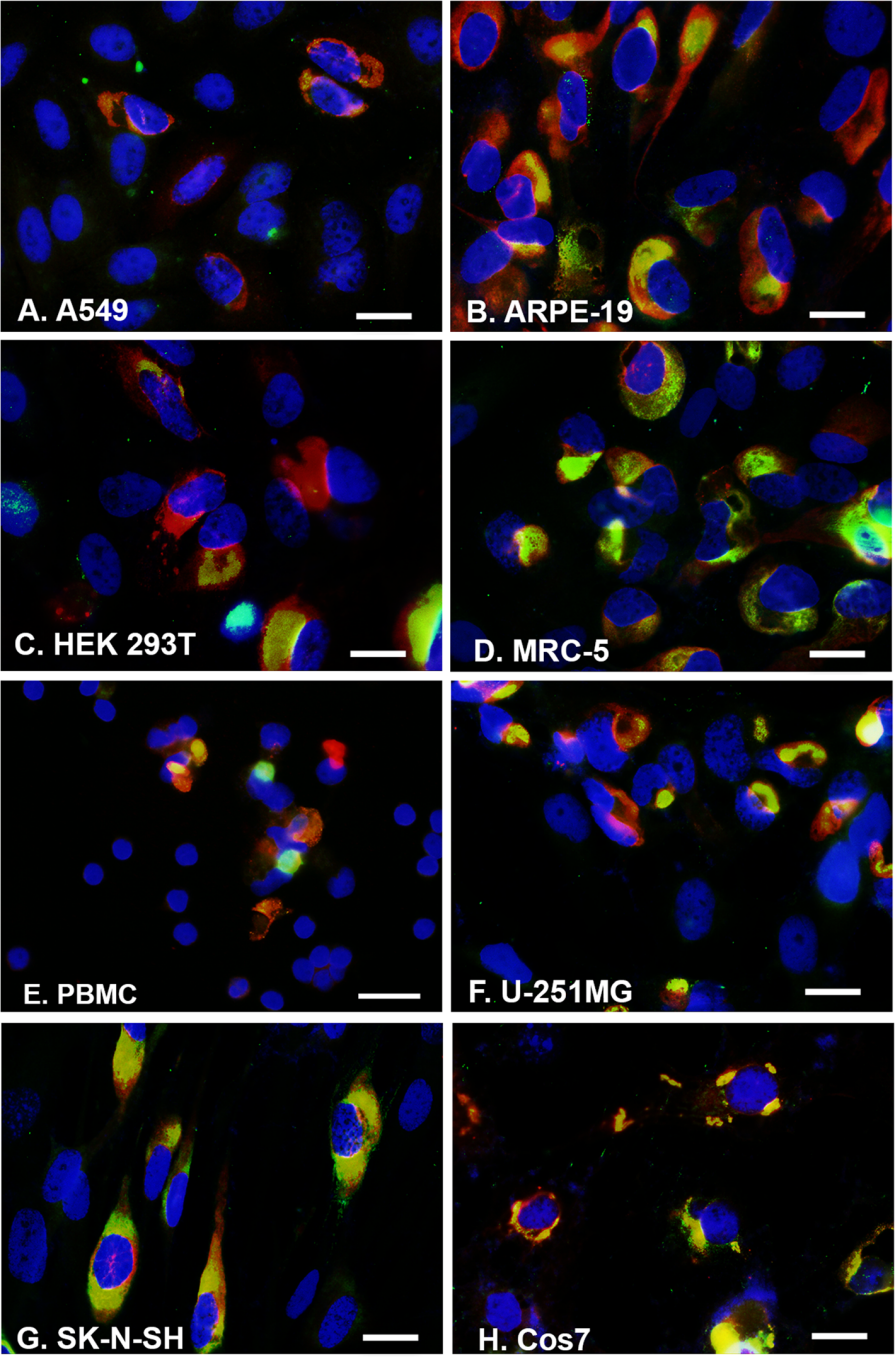
ZIKV RNA replication in different cell lines by RISH. ZIKV MR766 was used to infect different cell lines as indicated for 24 h at an MOI of 0.5. IFA was performed to show viral protein (*red*) and RISH was performed to show viral RNA replication (*green*). DAPI was used to show nuclei in *blue*. Scale bar: 10 μm. **a** A549, **b** ARPE-19, **c** HEK 293T, **d** MRC-5, **e** PBMC, **f** U-251MG, **g** SK-N-SH, **h** Cos7

Consistent with other reports [10, 11], the A549 cell (an epithelial cell line from lung cancer) was confirmed to be permissive for ZIKV infection (Fig .1a). We found that other epithelial cells including ARPE-19 (Fig. 1b) and HEK 293 T (Fig. 1c) are also permissive for ZIKV infection. In contrast, HEp-2 and HT1080 cells are not (or poorly) permissive for ZIKV infection. We tested the ZIKV infection in an endothelial cells (SLK). The endothelial cells did not support the ZIKV replication.

Inconsistent with another report [11], we found that ZIKV had different replication patterns in lines of human fibroblast cells. To examine the permissiveness of ZIKV in human fibroblast cells, we infected a human foreskin fibroblast (BJ, ATCC) and an HFF used in our laboratory previously for cytomegalovirus infection, and MRC-5 (human lung fibroblast). MRC-5 (Fig. 1d) are permissive for ZIKV infection, particularly the MRC-5 showed a better infection rate for ZIKV than Vero cells. However, the HFF in our laboratory and the BJ cell line from ATCC were not permissive for viral infection, which is different from the report from Hamel et al. that HFF was found to be completely permissive [11]. The permissiveness of ZIKV in human lung fibroblast cells (MRC-5) and in lung epithelial cell (A549) creates a new hypothesis that the ZIKV may infect and replicate in the human lung.

Mosquitos transmit ZIKV by direct contacting blood of the ZIKV carriers. We wondered whether ZIKV could grow in blood cells. We selected three kinds of blood source cells or blood cells (CEM/CD4 T cell, THP-1 monocyte, and PBMC). We found that PBMC is permissive for ZIKV infection (Fig. 1e), however the ZIKV cannot replicate in the T cell line and monocytes by the methods tested in our experiments. We, at this point, do not know which type of the blood cells are permissive for ZIKV infection. The observation that ZIKV infects PBMC is important because it suggests a mechanism for how ZIKV viremia is maintained. The maintenance of viral load in blood is important for ZIKV transmission by mosquitos [18].

The major medical problem caused by ZIKV infection is that it interferes with fetal development by causing microcephaly [19, 20]. ZIKV is infectious to the neural stem cells [10]. Here, we found that SK-N-SH, a neuroblastoma cell line that displays epithelial morphology, is also highly permissive for ZIKV infection (Fig. 1g). The effects of ZIKV infection on the stem cell proliferation has been studied for neural stem cells [10, 12, 21, 22], but it has not been reported from other type of cells in brain. We tested the permissiveness of ZIKV on a glioblastoma cell line (U-251MG) which is from a brain tumor. We found that U-251MG is highly permissive for ZIKV infection (Fig. 1f). The viral E protein and RNA production were shown to be comparable to these in Vero cells.

In addition, we verified that Cos7 cell line is permissive for ZIKV infection (Fig. 1h). Two cell lines from mouse (NIH3T3 and MEF-mouse embryo fibroblast) did not support ZIKV infection.

All the positively permissive cell lines are shown in Fig. 1, and the cell permissiveness is summarized in Table 1.

### RNA replication or viral protein production was further demonstrated in permissive cells by real-time RT-PCRs or western blots

**Fig. 2:**
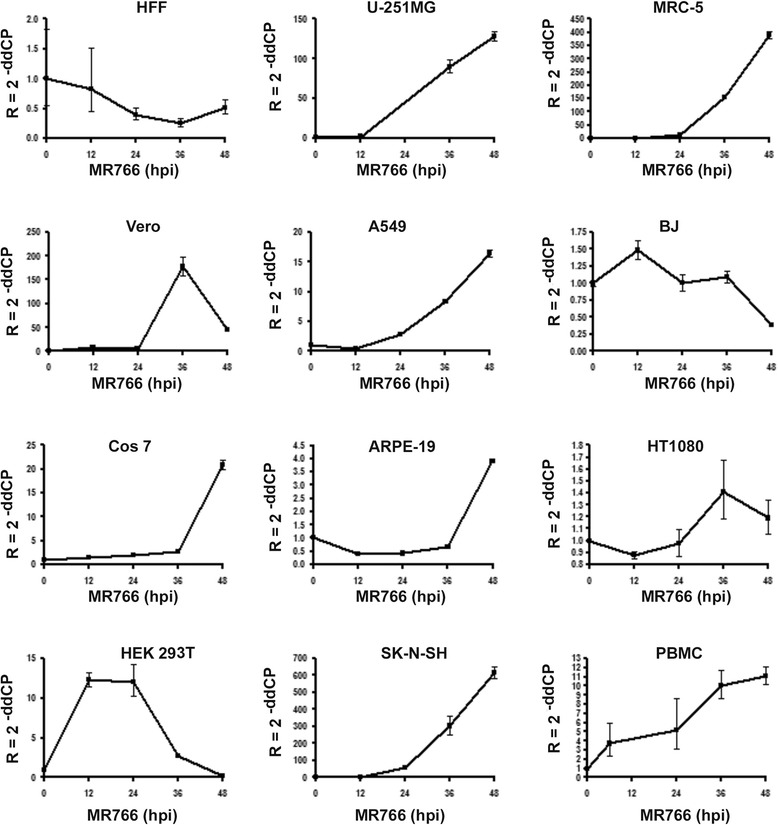
ZIKV RNA replication in different cell line by real-time RT-PCR. The cells as indicated were infected with ZIKV MR766 at an MOI of 0.1. The cells were collected at the time points as indicated the X axis for RNA isolation. The RNA samples were then applied for real-time RT-PCR using the primers as shown in the section of “Methods”

The RNA replication compartments shown by the RISH assay certainly shows viral RNA replication. To quantitatively determine the ZIKV RNA replication in real-time, we performed a real-time RT-PCR to examine the viral RNA level at different time points post infection in all kinds of the cells with an MOI of 0.1. The cell names are listed in the Table 1. We selected the results in a curve graph for all the permissive cells and the cells previously reported including HFF, Vero and A549. As can be seen in the Fig. 2, our real-time RT-PCR results are consistent with that of IFA and RISH studies (Table 1 and Fig. 1). A ZIKV RNA multiplication curve represents a RNA replication pattern and is defined as positive replication. ZIKV RNA level did not increase at all in HFF and BJ cells after infection overtime. In most permissive cells, viral RNA began to increase at time of 24 h after infection, but it started rising in U-251MG, A549 and SK-NSH cells at 12 hpi. In summary, we revealed that at least 7 different human cell lines (U-251MG, MRC-5, A549, ARPE-19, PBMC, SK-N-SH, and HEK293T) are permissive for ZIKV infection.

**Fig. 3:**
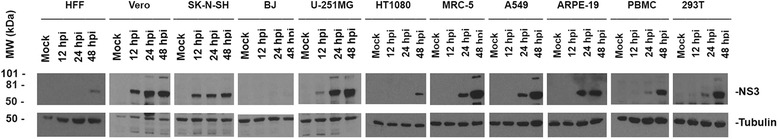
Western blot assay to examine the ZIKV protein production. ZIKV MR766 was used to infect the cell lines as indicated at an MOI of 0.5. The whole cell lysate (WCL) samples were collected at the time (hours post infection-hpi) as indicated on the top. Western blot assay was performed to examine the viral protein production using an anti-NS3 antibody. Tubulin was used as an internal sample loading control

In addition, we performed a western blot assay to examine the viral protein productions following the infection of ZIKV in each cell line at MOI of 0.5. The samples were collected at the indicated time after infection (mock, 12 hpi, 24 hpi, and 48 hpi as shown in Fig. 3). After separated in a gradient PAGE gel (ThermoFisher Scientific.), the protein was tranfered to a membrane for blotting with anti-NS3 antibody. As can be seen, our results of western blot assay are consistent to those of real-time RT-PCR. ZIKV is more infectious to Vero, SK-N-SH, U-251MG, MRC-5 and A549 than to PBMC, 293 T, HT1080 and ARPE-19. Its infection in HFF or HT1080 only produced very weak protein at 48 hpi that is not detectable in another human foreskin fibroblast cell line, BJ. Therefore, we identified 7 more human cell lines that are permissive for ZIKV infection.

### Viral protein is detected ahead of viral replicative RNA by IFA and RISH

Generally, at the early time of viral infection, some viral proteins need to interact with cellular proteins to form a restricted compartment that is utilized by the the virus for viral DNA/RNA replication. For most RNA viruses, RNA replication occurs in the cytoplasm [23]. If proteins of ZIKV are important for viral RNA replication and forms a pre-replication compartment, the viral proteins should be produced before RNA replication occurs. To demonstrate the presumption, we performed an IFA to examine viral E protein production and a RISH to examine viral RNA replication with a time course of infection in Vero cells.

**Fig. 4:**
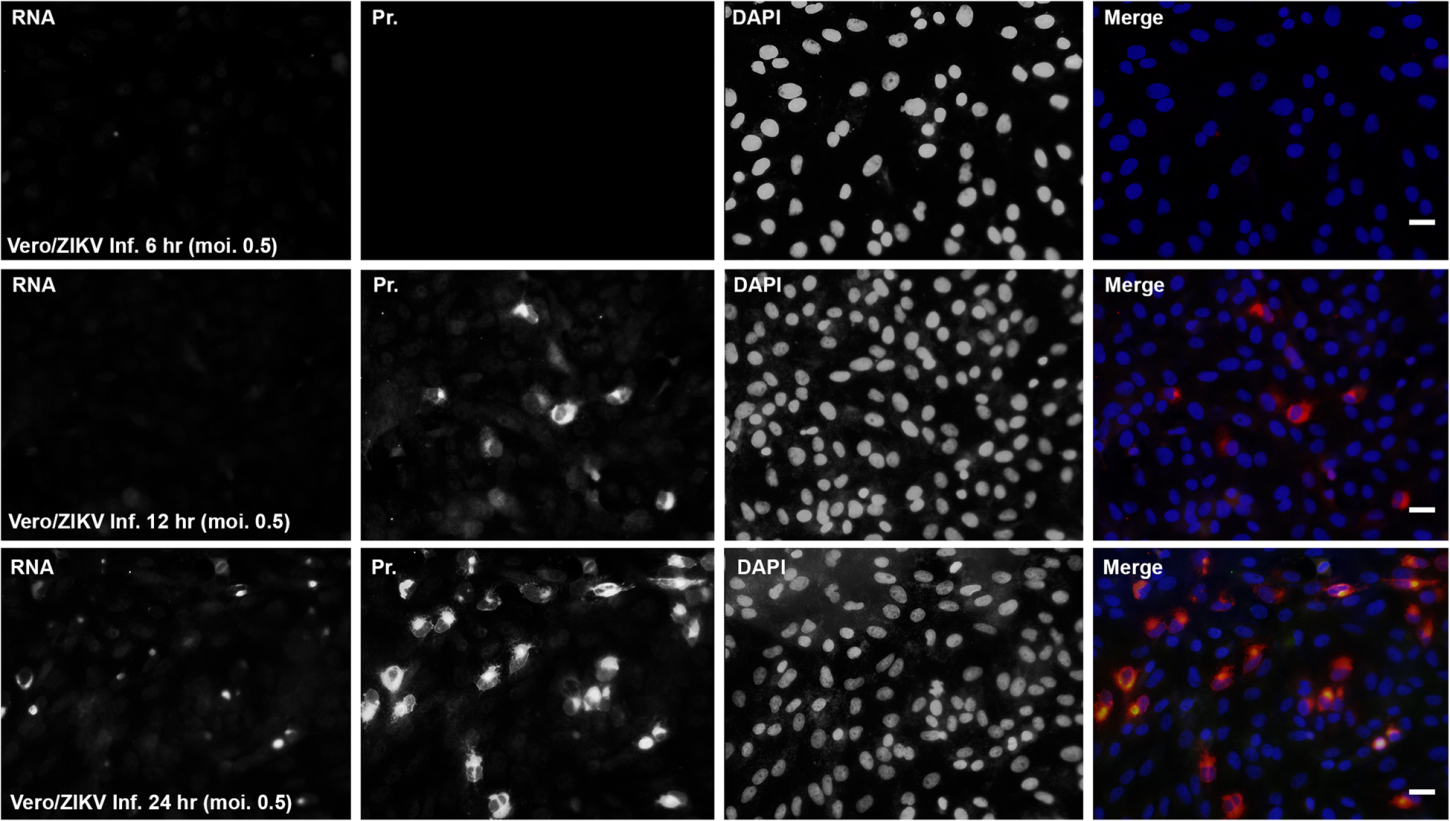
Viral protein was produced ahead of viral RNA replication. Vero cells were infected with ZIKV MR766 at an MOI of 0.5. The cells were fixed at 6, 12 and 24 hpi. IFA and RISH were performed to show the production of viral protein and RNA. In the merged panels, RNA was shown in *green*, viral protein was in *red*, and DAPI was used to show nuclei in *blue*. Scale bar: 10 μm

As shown in Fig. 4, we infected Vero cells with ZIKV MR766 at an MOI of 0.5. The cells were fixed at 6, 12 and 24 hpi. To show the overlapping of viral E protein, RNA, and nuclei, the images were merged. In the merged panels, RNA was shown in green, viral E protein was in red, and DAPI was used to show nuclei in blue. At 6 hpi, neither viral E protein nor viral RNA was detected. At 12 hpi, viral E protein was clearly produced in many cells but viral RNA was not able to be seen. At 24 hpi, more cells were positive with the E protein staining than that at 12 hpi. Both viral E protein and RNA were detected at this time point, and viral RNA replication occured in the E protein-formed domains. Therefore, we revealed that viral E protein is produced before viral RNA replication. The results also imply that the viral E protein may be important for viral RNA replication.

### Viral E protein directly interacts with viral RNA

**Fig. 5:**
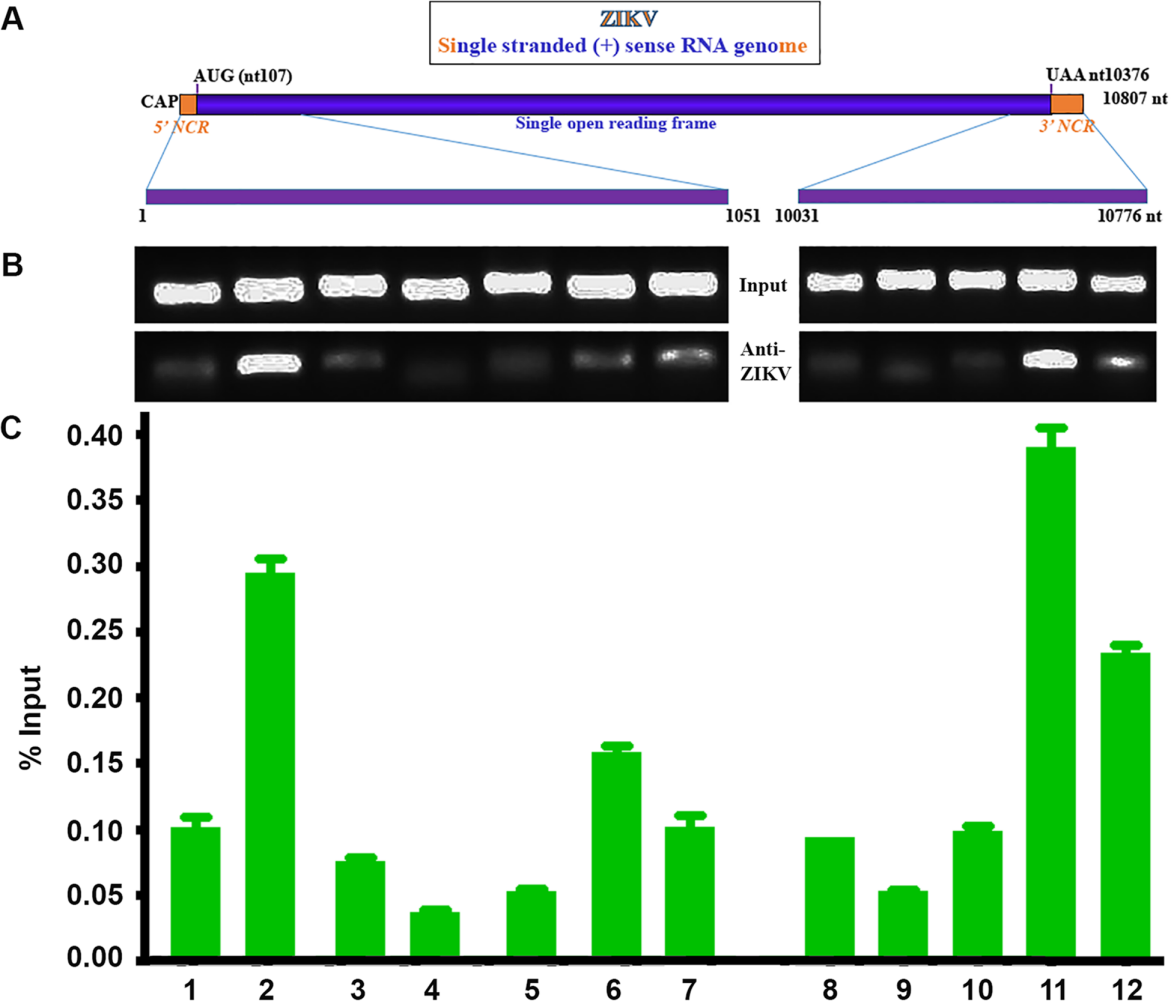
RNA chromatin immunoprecipitation (ChIP) assay to determine the interaction of ZIKV protein with viral RNA. **a** The diagram of ZIKV genome and the area covered by the primers listed in Table 2: 5′ side, from 1 to 1051; 3′ side, from 10,031 to 10,776. **b** RNA ChIP assay examined by regular RT-PCR. The Vero cells were infected with ZIKV MR766 at an MOI of 0.5 for 24 h. Then the cells were fixed with 4% formaldehyde. The cells were then sonicated to shear the RNA to 250–500 nt. The samples were then divided into 3 part: 1) input, 2) normal mouse IgG-precipitation, and 3) anti-ZIKV antibody precipitation. In both 2) and 3), the RNA-protein complex was pulled down using an anti-ZIKV mouse serum generated from a ZIKV-infected mouse or the normal mouse IgG (as negative control) with the beads. After washing for 3 cycles, the protein was digested with proteinase K, and the DNA was removed by DNase I (RNase free). The input RNA and the ChIPed RNA samples were then precipitated and the samples were used for RT-PCR. The PCR products were applied to run an agarose gel and visualized. **c** RNA ChIP assay examined by real-time RT-PCR. The same as B, but used a real-time RT-PCR. The yields of PCR from ChIPed RNA or from input RNA were normalized to IgG. The ratio of PCR products from ChIPed RNA were then compared to the input shown as % Input

As we saw that the E protein always overlapped with viral replicative RNA in our IFA and RISH experiment, we wondered whether the E protein interacts with viral RNA. An interaction between the protein and viral RNA always suggests the importance of the viral protein in viral RNA replication. To confirm the interaction of viral RNA with viral protein, we employed an RNA ChIP assay. For so doing, we designed 2 sets of primers that continuously cover the 1051 nt at the 5′ side of the genome and 745 nt at its 3′ side, respectively (shown in Fig. 5a). To design the primers, we considered to have 15–20 nt overlapping between the neighboring PCR fragments and the resultant PCR fragments are 150–180 bp. The sequences and positions of the primers are summarized and listed in Table 2.

After infected with ZIKV MR766 for 24 h at an MOI of 0.5, the Vero cells were fixed and sonicated to shear the viral genome to a range of 250–500 nt. The RNA-protein complexes were then pulled down by anti-ZIKV antibody-conjugated beads. After careful washing, the beads were then eluted, and the eluate (containing RNA, DNA, and proteins) was treated with proteinase K and DNase I (RNase free). The RNA was finally precipitated and used for RT-PCR. We performed the end point RT-PCR (Fig. 5b) and real-time RT-PCR (Fig. 5c). As can be seen in Fig. 5b, the PCR products showed a strong band in the 2nd, 6th and 11th lanes. The 2nd (nt 135–294) and 6th (nt 734–899) lanes respond the nucleotides within the first 1051 nt of viral genome, while the 11th (nt 10,474–10,644) lane reflects the nucleotides in the 3′ UTR of viral genome. The real-time RT-PCR shown in Fig. 5c is consistent with the results of the regular RT-PCR: the 3 peaks are correspondent to the 3 stronger bands seen in the Fig. 5b. Therefore, our RNA ChIP assay results demonstrated that the viral E protein interacts with viral replicative RNA. At least three E protein-binding sites were identified in the ZIKV MR766 RNA genome.

### ZIKV RNA co-localizes with ER, and is probably associated with Golgi apparatus and mitochondria

**Fig. 6:**
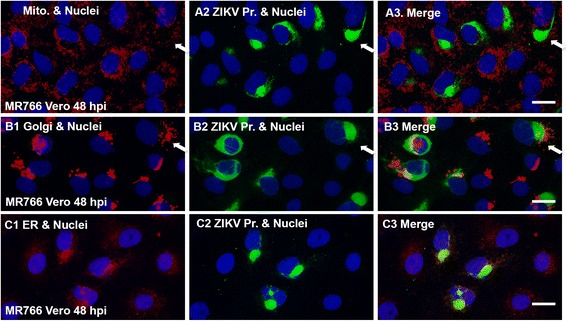
Association of ZIKV RNA replication with cellular organelles. Vero cells were infected with MR766 at an MOI of 0.5 for 24 h. The cells were then fixed for immunostaining in red using anti-Cox IV antibody for showing mitochondria (A1-A3), anti-Giantin antibody for visualizing Golgi apparatus (B1-B3), and anti-Calreticulin for examining ER (C1-C3). Then the cells were hybridized with ZIKV-specific probe to show viral replicative RNA in green. DAPI was used to show nuclei in blue. Scale bar: 10 μm

The ER, mitochondria, and Golgi apparatus are cytoplasmic organelles with special biological functions. Since flaviviruses, containing positive single strand RNA genome, replicate in the cytoplasm, we wondered whether ZIKV RNA replication could associate with any of the cytoplasmic organelles. For doing so, we infected Vero cells with MR766 at an MOI of 0.5 for 48 h. The cells were then fixed for immunostaining in red using anti-Giantin antibody for visualizing Golgi apparatus, anti-Cox IV antibody for showing mitochondria, and anti-Calreticulin for examining ER. Then the cells were hybridized with ZIKV-specific probe to show viral replicative RNA in green. As can be seen in Fig. 6, the replicated ZIKV RNA colocalizes with ER (C1-C3). Although the replicated RNA is not overlapping with the mitochondria and Golgi apparatus, the mitochondrial and Golgi proteins are surrounding the RNA replication domains as shown by the arrows (A1-A3 and B1-B3). Therefore, the cytoplasmic apparatus may play important roles in ZIKV RNA replication.

## Discussion

During the evolution of the ZIKV, it appears that the ZIKV underwent a pathogenic differentiation: Asian lineage ZIKV has been linked to recent epidemics and high likely the congenital microcephaly and GBS while African strains cause milder symptoms. However, how the ZIKV infection results in the neural diseases is not fully understood. The knowledge of the molecular biological interactions of the ZIKV with host are lacking, although it is critical to develop anti-ZIKV strategies. Although it is believed that ZIKV infection can be transmitted via *Aedes* species mosquito biting and/or sexually, other transmission route may exist [24]. Viral transmission is related to cell permissiveness for infection. Here, we performed comparative studies of cell permissiveness for ZIKV African strain (MR766) and Asian strain (PRVABC59). Both strains infect a wide range of human cell types. No significant difference was found in terms of RNA replication and viral protein production in those cells between the two strains. Further studies are needed to figure out whether they have differences in viral RNA replication and infection at in vivo level.

Viral permissiveness may relate viral replication to viral transmission and spreading. For example, one of the spreading routes of ZIKV is by *Aedes* mosquito biting [6, 7]. The most substance the mosquito obtains from the ZIKV-carrying host is blood, which is also the source of mosquito infection. We tested endothelial cell for the infection of ZIKV and found that the endothelial cells are not permissive for ZIKV infection. Importantly, our IFA, RISH, and real-time RT-PCR experiments demonstrated that ZIKV productively infects the PBMC (Figs. 1 and 2, Table 1). Although we do not know yet which type of blood cells are permissive for ZIKV infection, our finding that ZIKV infects PBMC suggests that PBMC is the source of blood virus and is important for maintenance of viral level in the blood.

It has been reported that a great number of viral particles of ZIKV were detected in brain tissue and fluid [25]. It has been reported that ZIKV productively infects neural stem cell [10, 12, 21, 22]. Another important cell line that supports ZIKV infection is U-251MG (Figs. 1 and 2, Table 1). U-251MG is a glioblastoma cell line and derived from brain. Our results showed that ZIKV replicates in U-251MG productively, which may suggest another factor that enhances the ZIKV pathogenesis in brain. Further studies using a mouse model will be necessary to confirm our hypothesis that ZIKV infects different types of cells in brain tissue to affect the development of the fetal brain.

ZIKV is a member of the family *Flaviviridae*. It contains a positive and single stranded RNA genome. Viral protein can be produced immediately after viral entry, which is consistent with our finding that viral protein was detected before viral RNA replication can be seen (Fig. 4). Flaviviral genome replication requires three steps: 1) negative strand RNA synthesis, 2) positive strand RNA synthesis, and 3) 5′-RNA capping and methylation. Before viral RNA replication, a pre-replication complex needs to be formed [26, 27]. The pre-replication complex contains both viral and cellular proteins. Some of these proteins may be able to bind to viral RNA. Our RISH and IFA results showed that the viral replicative RNA localizes in the cytoplasm as a domain that overlaps with viral E protein, forming the viral RNA replication domain (Fig. 1). In this study, RNA ChIP assays demonstrated that viral proteins directly interact with viral RNA (Fig. 5). At least 3 binding sites have been identified in the viral genome for the viral E protein binding (Fig. 5). This results is surprising because E protein is a structural protein of ZIKV and functions as an initiator of viral infection through interacting with cell receptor. However, we found that E protein presents continuously in the viral replication compartments. The finding that E protein interacted with viral RNA implies that E protein may play roles in viral replication. This is important because the protein-RNA interaction may be an effective target for designing the intervention drugs against viral RNA replication.

Lastly, we revealed that the viral replication occurs in ER (Fig. 6C1–C3), which is consistent with the report for other flaviviral RNA replication [26, 27]. Although the RNA replication compartment is not colocalizing with mitochondria or Golgi apparatus, the matrix protein of mitochondria is shown surrounding the RNA domain as shown by the arrow in the Fig. 6A1-A3. A similar observation was also shown for Golgi apparatus in the Fig. 6B1-B3. This information suggests that the cellular organelles may play different roles in ZIKV viral replication.

In summary, we employed different techniques to determine the ZIKV infection permissiveness in human cells, the two strains have a similar permissiveness in human cells. More experiments are needed to reveal the differences between Asian and African strains because it is important to know why only the strains derived from Asian linage cause microcephaly. We revealed that ZIKV can replicate to a high titer in human lung fibroblast cells, which imply that ZIKV can replicate in the lung. PBMC is permissive for ZIKV replication, which is important because the information may explain why the mosquito is the transmitter for ZIKV. We also discovered that viral protein directly interacts with viral genome via binding to different sites of the viral genome. We will test whether the protein-RNA interaction can be a target for designing drugs to intervene the viral replication and infection.

## Conclusions

Our results that E protein of ZIKV interacts with viral RNA imply that the E protein may be important for viral RNA replication. Our results provide not only the information of ZIKV permissiveness that guides the usage of human cells for the ZIKV studies, but also the insight into the viral RNA-E protein interaction that may be targeted for intervention by designing small molecule drugs. Our future studies will be focused on how the ZIKV infection permissiveness is determined by virus-host interactions.

## Additional file

Additional file 1: Figure S1. Immunofluorescent assay and RISH to show ZIKV viral RNA replication and protein production in Vero cells. Vero cells were infected with MR766 (A1-D1, A3-D3) or PRVABC59 (PR) (A2-D2, A4-D4) at an MOI of 0.5 for 24 h. After fixation with 1% formaldehyde, the cells were permeabilized and immunostained for viral protein in red (B1-B4). After refixation with 4% formaldehyde, the cells were hybridized with single strand DNA probe (labeled with biotin) made from ZIKV genomic DNA. The RNA was stained in green (A1-A4). The cell nuclei were shown in blue using DAPI staining (C1-C4). The merged image was shown in D1-D4. In the panels 1 and 2, the pictures were taken using a 40× lens. In the panels 3 and 4, the pictures were taken using an 100× lens. Scale bar: 10 μm. (PSD 5413 kb)

## Abbreviations

ChIP: Chromatin Immunoprecipitation
ER: Endoplasmic reticulum
GBS: Guillain–Barré syndrome
IFA: Immunofluorescence assay
NS proteins: Non-structural
PBMC: Peripheral blood mononuclear cell
RISH: RNA in situ hybridization
ssRNA: single stranded RNA
WCL: Whole cell lysate
ZIKV: Zika virus
